# Unraveling the hierarchical genetic structure of tea green leafhopper, *Matsumurasca onukii*, in East Asia based on SSRs and SNPs

**DOI:** 10.1002/ece3.9377

**Published:** 2022-10-01

**Authors:** Li Zhang, Christopher H. Dietrich, Ye Xu, Zhaofu Yang, Maohua Chen, Thai H. Pham, Cuong C. V. Le, Li Qiao, Masaya Matsumura, Daozheng Qin

**Affiliations:** ^1^ Key Laboratory of Plant Protection Resources and Pest Management of the Ministry of Education, Entomological Museum Northwest A&F University Yangling Shaanxi China; ^2^ Institute of Jiangxi Oil‐Tea Camellia, Jiujiang University Jiujiang Jiangxi China; ^3^ Illinois Natural History Survey Prairie Research Institute, University of Illinois Champaign Illinois USA; ^4^ College of Agriculture, Jiangxi Agricultural University Nanchang Jiangxi China; ^5^ Mientrung Institute for Scientific Research, Vietnam National Museum of Nature, VAST Hue Vietnam; ^6^ Graduate School of Science and Technology, Vietnam Academy of Science and Technology Hanoi Vietnam; ^7^ College of Agronomy, Xinyang Agricultural and Forestry University Xinyang Henan China; ^8^ Institute for Plant Protection, National Agriculture and Food Research Organization Tsukuba Ibaraki Japan

**Keywords:** *Matsumurasca onukii*, reduced‐representation genome sequencing, SNPs, SSRs, subtle genetic differentiation, tea pest

## Abstract

*Matsumurasca onukii* (Matsuda, R. (1952). *Oyo‐Kontyu Tokyo*, **8**(1): 19–21), one of the dominant pests in major tea production areas in Asia, currently is known to occur in Japan, Vietnam, and China, and severely threatens tea production, quality, and international trade. To elucidate the population genetic structure of this species, 1633 single nucleotide polymorphisms (SNPs) and 18 microsatellite markers (SSRs) were used to genotype samples from 27 sites representing 18 geographical populations distributed throughout the known range of the species in East Asia. Analyses of both SNPs and SSRs showed that *M. onukii* populations in Yunnan exhibit high‐genetic differentiation and structure compared with the other populations. The Kagoshima (JJ) and Shizuoka (JS) populations from Japan were separated from populations from China by SNPs, but clustered with populations from Jinhua (JH), Yingde (YD), Guilin (GL), Fuzhou (FZ), Hainan (HQ), Leshan (CT), Chongqing (CY), and Zunyi (ZY) tea plantations in China and the Vietnamese Vinh Phuc (VN) population based on the SSR data. In contrast, CT, CY, ZY, and Shaanxi (SX) populations clustered together based on SNPs, but were separated by SSRs. Both marker datasets identified significant geographic differentiation among the 18 populations. Various environmental and anthropogenic factors, including geographical barriers to migration, human transport of hosts (*Camellia sinesis* [L.] O. Kuntze) and adaptation of *M. onukii* to various local climatic zones possibly account for the rapid spread of this pest in Asia. The results demonstrate that SNPs from high‐throughput genotyping data can be used to reveal subtle genetic substructure at broad scales in r‐strategist insects.

## Graphical abstract

In this study, SNPs and microsatellite markers were used to capture multiscale patterns of genetic structure of *Matsumurasca onukii*, one of the dominant pests in major tea production areas in Asia. General migration route showed that there are two diffusion sources including Yunnan and the other tea plantations of China, respectively, from where introduced to Japan tea plantations. Our findings indicate that the host plants and geographical distances are significant involved to the genetic variation of *M. onukii* between different Clusters and that SNPs from high‐throughput genotyping data can be used to reveal subtle genetic substructure at broad scales in r‐strategist insects.

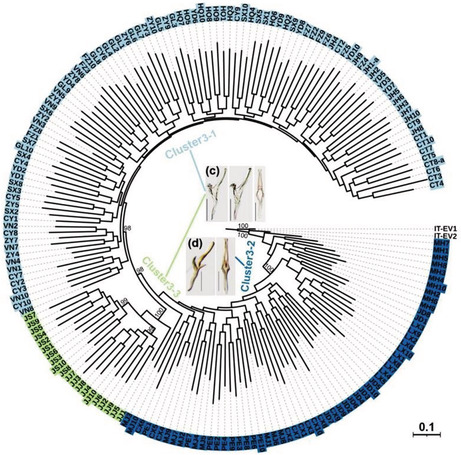

## 1 INTRODUCTION

Recent methodological advances in population genetic and data analysis have greatly facilitated our understanding of genetic diversity, population structure, and microevolution of insect pests, which can guide the prediction of evolutionary potential (such as environmental adaptability and population fecundity) and facilitate ecological regulation and management (such as biocontrol technology and pest behavior regulation) (Roderick, 1996; Roderick & Navajas, 2003). However, detecting subtle genetic differentiation remains a major challenge because many species, particularly r‐strategists, have large effective population sizes and high gene flow due to long‐distance dispersal or human‐mediated movement on crop plants (Matsumoto et al., 2013; Mun et al., 1999; Shi et al., 2012). In addition to exhibiting subtle levels of genetic differentiation, such populations may display diverse patterns of genetic structure, including classical patterns of isolation by vicariance, host, regional clustering, origin, and dispersion (Du et al., 2020; Goodman et al., 2019), as well as complex patterns that lack clear spatial structuring or that may be influenced by anthropogenic factors and habitat (Jiang et al., 2020; Kareem et al., 2018; Raszick et al., 2019). Identifying subtle complex genetic patterns remains a critical objective for developing effective control measures because some pest management strategies require detailed knowledge of genetic diversity and genetic structure determination that can underpin downstream efforts to measure contemporary and historical migration, delineate groups within populations, and infer evolutionary history (Jiang et al., 2020; Raszick et al., 2019).

Tea green leafhopper, *Matsumurasca onukii* (formerly *Empoasca onukii*), is one of the dominant pests in the major tea production regions of East Asia (Chen et al., 2020; Fu, Han, & Xiao, 2014; Qin et al., 2015), the species was originally described by Matsuda (1952) as an injurious tea leafhopper in Japan. Although this species was also observed in Chinese tea plantations since the 1960s, it was widely misidentified as “*Empoasca vitis*” until recently (Kuoh & Zhang, 1988; Lv et al., 1964; Zhang, 1980; Zhao et al., 2000). Recent clarification and phylogenetic analysis of the tribe Empoascini confirmed that the Chinese and Japanese populations are conspecific and correctly named *Matsumurasca onukii* (Dworakowska, 1971, 1982; Fu, Han, & Xiao, 2014; Qin et al., 2015; Xu et al., 2021). Previous comparative morphological studies revealed obvious intraspecific variation among populations, with individuals from different populations in southern China differing in the shape of membranous flanges and lengths of spiny protuberances on the shaft of the male aedeagus (Qin et al., 2015; Xu et al., 2021). This pest currently is widely distributed and causes considerable yield loss in diverse cultivated varieties of *Camellia sinesis* (L.) O. Kuntze in Japan, Vietnam, and China that are grown under a variety of ecological conditions (Dworakowska, 1971, 1982; Qin et al., 2015). Based on these prior morphological observations, the broad distribution of the species and the differences in environmental conditions among tea‐growing regions, we hypothesized that these morphological variations reflect genetic differences among geographic populations.

Previous studies of the population structure and genetic differentiation of tea green leafhopper have used mitochondrial gene sequence data or limited numbers of microsatellite markers (Chen et al., 2015; Fu, Li, et al., 2014; Li et al., 2013; Zhang et al., 2016, 2019; Zhang et al., 2018; Zhou et al., 2014; Zhu et al., 2019). Microsatellite‐based results revealed some geographical differentiation between populations, particularly between two populations in Yunnan and the other Chinese populations but provided only limited evidence of consistent population genetic structure, potentially affected by endogenous properties of the markers used (e.g., fluctuations in allele frequency and recombination rates), the quality of genetic data and the sampling methods. For the present study, we obtained more extensive data using next‐generation sequencing (NGS) to more fully characterize the genetic substructure in *M. onukii* population in East Asia.

The NGS methods based on reduced‐representation genome sequencing (RRGS) have become widely used in population genetic analyses, particularly genotyping‐by‐sequencing (GBS) and restriction‐associated DNA sequencing (RAD‐seq) (Andrews et al., 2016; Davey et al., 2011; Elshire et al., 2011). These methods allow rapid detection of novel nuclear markers at the minimal cost. Genome‐wide SNPs can provide more power for understanding population dynamics and pest species than mitochondrial DNA or microsatellite (SSR) markers (Emerson et al., 2010; Liu et al., 2019). While SSR (e.g., high polymorphism) can provide estimates of allelic distribution across whole populations, SNPs can compensate for the drawbacks of SSRs (e.g., homoplasy, complex mutation pattern, and high prevalence of null allele) (Coates et al., 2009; Kraus et al., 2015; Morin et al., 2004). Analysis of hundreds to thousands of SNPs per population has also allowed for the use of more sophisticated tools to address questions of genetic differentiation, complex patterns, and connectivity between populations (Dowle et al., 2017; Havill et al., 2017, 2019; Ma et al., 2020).

In the present study, microsatellite markers were coupled with high‐throughput sequencing approaches to obtain three goals: (i) to assess the spatial distribution of genetic variation of *M. onukii* populations in East Asia based on the microsatellite markers, followed by more detailed analysis of genetic differentiation using two types of molecular markers; (ii) to compare population relationships and levels of geographic differentiation among populations from the performances of SNPs and microsatellite markers and determine if patterns reflect morphological variation among populations; and (iii) to estimated historical migration between genetic clusters for inferring the evolutionary history of *M. onukii* populations in East Asia. This study will reveal correlated patterns of morphological variation and hierarchical genetic structure in East Asian *M. onukii* population, which will be useful in monitoring species distribution and future population dynamics. This will be enable better prediction, regulation, and management of *M. onukii* populations in different tea producing regions of East Asia.

## 2 MATERIALS AND METHODS

### 2.1 Sampling and species identification

**FIGURE 1:**
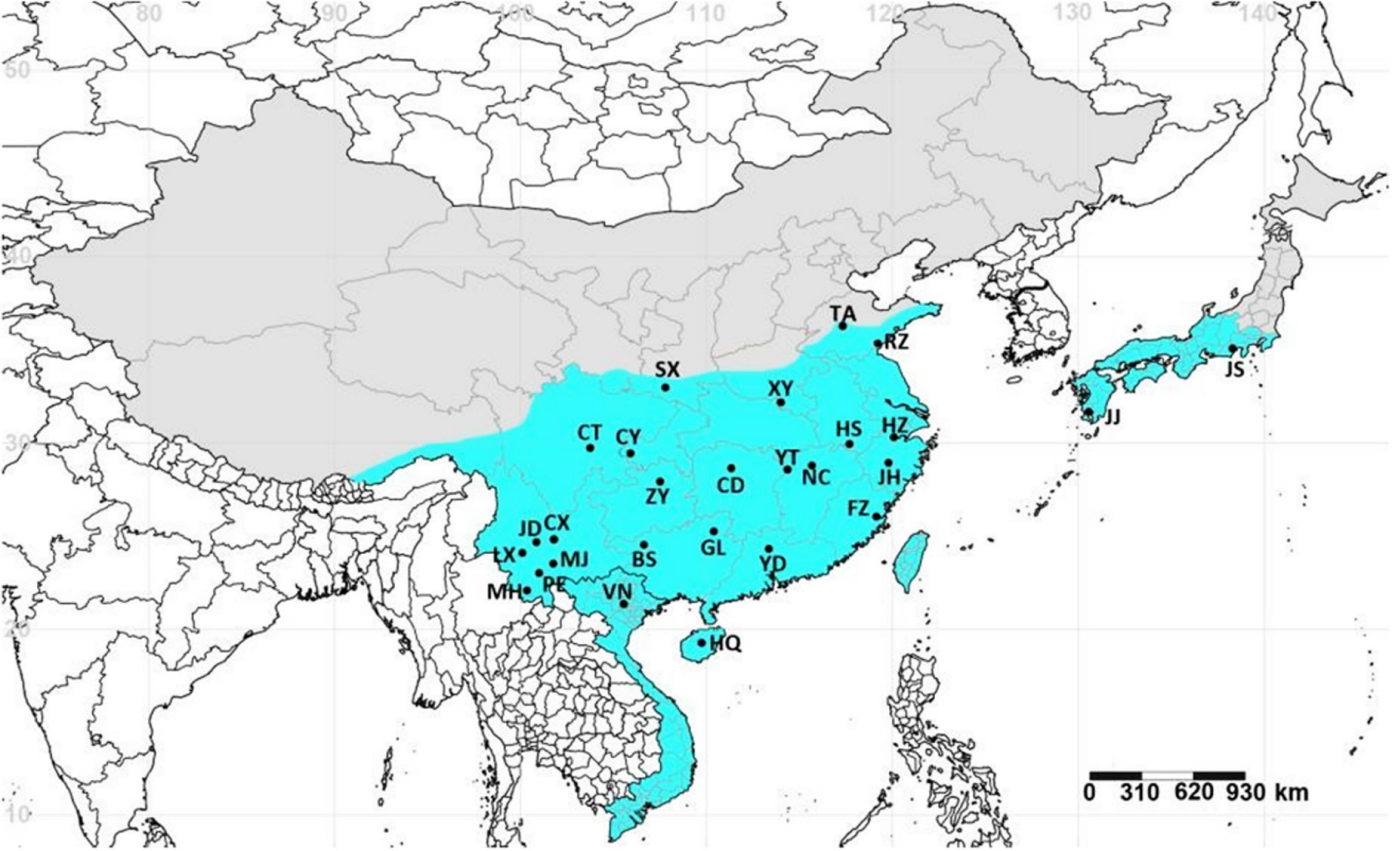
Geographic distribution and populations of *Matsumurasca onukii*. Population codes are listed in Table S1. SimpleMappr was used to produce a distribution map based on the geographical coordinates in Table S1. http://www.simplemappr.net/#tabs=0.

Tea green leafhopper specimens of 27 representative populations, including 24 Chinese localities, two populations from Japan (Kagoshima and Shizuoka), and one population from Vietnam (Vinh Phuc), were collected during the seasons of tea green leafhopper occurrence from 2013 to 2020 (Table S1). The samples included specimens collected for our previous study as well as additional samples from tea producing areas of Japan (Shizuoka [JS]), Vietnam (Vinh Phuc [VN], Hainan [HQ]) and Southwest China (Mojiang [MJ], Menghai [MH], Lincang [LX], and Jingdong [JD]) (Table S1; Figure 1). All the collection sites were areas of more than 6 ha under continuous tea cultivation without weeds or trees. Adult specimens were captured by sweep net in five collecting points (10 m^2^) near the center of each site, spaced 100 m or more apart. Specimens from all collecting sites within a location were pooled to represent one population. All these specimens were preserved in 100% ethanol and stored in the Entomological Museum, Northwest A & F University, Yangling, China (NWAFU).

Species identifications were confirmed by examining the morphological characters of all specimens from each representative population by dissection under an OLYMPUS SZX‐10 Stereoscopic Zoom Microscope. Male genitalia of each specimen was separately preserved in a small tube prefilled with glycerin and diagnostic characters (especially aedeagus characteristics) among populations were checked and compared to confirm the species identification and check for intraspecific variation.

### 2.2 DNA extraction

Thirty male adults in each population, except for the dissected male genitalia used for species identification, were used for extracting DNA using either the CTAB method or DNeasy blood and tissue kits (Qiagen). The quality of DNA was detected and quantified with a Nanodrop One (Thermo Scientific).

### 2.3 Genetic structure using SSRs

#### 2.3.1 SSRs amplification and genotyping

Individuals of seven populations (JS, VN, HQ, MJ, MH, LX, and JD) were genotyped using 18 previously developed microsatellite markers (Table S2; Zhang et al., 2016). Genotypes of the other 20 populations were obtained from previous studies (Zhang et al., 2019). All forward primers were labeled with fluorescent markers (FAM, HEX, and TAMRA). DNA from each individual was amplified for these markers in a 10 μl volume. PCR reactions followed the protocol described by Zhang et al. (2016). All the products were pooled and genotyped by automated capillary electrophoresis on an ABI 3130xl at Sangon Biotech. Microsatellite alleles were scored using the Microsatellite Plugin v.1.4 in Geneious v. 7.1.7 (Biomatters Ltd). The fragment lengths of different loci in different populations were calculated according to the molecular internal reference (Liz 500). All the genotypic data conversions were performed with GenALEx v. 6.5 (Peakall & Smouse, 2012) and CONVERT (Glaubitz, 2004).

#### 2.3.2 Population genetic structure analysis

The frequency and signs of null alleles were investigated using FreeNA (Chapuis & Estoup, 2007) and Micro‐Checker (Van Oosterhout et al., 2004). Tests for the Hardy–Weinberg Equilibrium (HWE) and Linkage disequilibrium (LD) were carried out using Genepop v. 4.2. The frequencies of alleles and allelotypes, and allelic richness (AR) were calculated using Genepop v. 4.2 (Rousset, 2008), Cervus v. 2.0 (Marshall et al., 1998), and Fstat v. 2.9.4 (Goudet, 2003), respectively. Effective number of alleles (N_E)_, observed heterozygosity (H_O_), and expected heterozygosity, (H_E_) were determined using GenALEx v. 6.5.

Structure v 2.3.4 was used to estimate the number of genetically distinct clusters (K) among the *M. onukii* populations using an admixture ancestry model with a burn‐in period of 5 × 10^4^ iterations and 10^6^ Markov Chain Monte Carlo (MCMC) repetitions. The range of possible clusters was set from 1 to 27 or to 18, with 20 independent runs for each K (Pritchard et al., 2000; Pritchard et al., 2010). The optimal K‐values in *M. onukii* populations was estimated by posterior probability of the data (L (K)) and the ad hoc statistic (ΔK) using the Web server Clumpak was used to align results from replicate analyses and visualize population structure (Evanno et al., 2005; Kopelman et al., 2015; Pritchard et al., 2000; Pritchard et al., 2010). Principal component analysis (PCoA) was performed by GenALEx v. 6.5. Neighbor‐Joining (NJ) tree based on Nei's genetic distance (*D*_*A*_) was constructed using POPTREE v. 2.0 (Takezaki et al., 2010), and bootstrap values were obtained using 1000 sample replicates. The analysis of molecular variance (amova) based on population clustering from Structure v. 2.3.4 and *F*_ST_ was calculated using Arlequin v. 3.11 (Excoffier & Lischer, 2010).

### 2.4 Genotyping by sequencing (GBS)

#### 2.4.1 Library construction for SNP sequencing

Based on the SSR population genetic analysis results, one to six populations (each population including 10 males) with significant genetic differentiation were selected from four genetic clusters. The GBS libraries were constructed and sequenced following the protocol of Elshire et al. (2011) and Onyango et al. (2016) with modifications.

*Ape*KI and 300–600 bp fragments were determined by detecting genome size and its restriction sites. DNA (more than 500 ng) extracted from one sample of *M. onukii* from each of the 18 populations was added to 96‐well plates with 3 μl 10 × NEB buffer and 2 μl *Ape*KI (5 U/μl) (reagents from New England Biolabs) to a final volume of 30 μl and heated in water for 2 h. Following ligation, samples in PCR‐96‐well plates in 40 μl with 60 picomoles barcode DNA (5′‐ACACTCTTTCCCTACACGACGCTCTTCCGATCTxxxx and 5′‐CWG yyyyA GATCGGAAGAGCGTCGTGTAGGGAAAGAGTGT) and universal adaptor (5′‐CWGAGATCGGAAGAGCGGTTCAGCAGGAATGCCGAG and 5′‐CTCGGC ATTCCTGCTGAACCGCTCTTCCGATCT), 1 mM dNTPs, 5 units T4 DNA Ligase and 4 μl10 × T4 DNA Ligation Buffer. The reaction system was incubated for 1 h and inactivated for 30 min at 65°C. The Barcode DNA provided special identification tags for each sample, and the included universal adaptor that also provided binding sites for sequencing primers. All the DNA fragments (300–600 bp) were pooled in a sequencing library after purification and recovery. Following amplification with sequencing primers, purification, and recovery, the quality of the library was assessed using an Aglient 2100 Bioanalyzer, KAPA Library Quantification Kit and Qubit 2.0. The libraries were then sequenced on an lllumina HiSeq 2500 platform using a 150 bp Paired End protocol at Allwegene Technologies.

#### 2.4.2 SNP genotyping

Raw reads were obtained by Base calling, assessed for quality, and filtered by length of fragment, Q‐value, and sequence alignment in databases. The identification of SNPs in Raw reads was accomplished using the Universal Network Enabled Analysis Kit (UNEAK) in TASSEL 3.0 (Bradbury et al., 2007; Lu et al., 2012, 2013). First, these reads were assigned to different individuals and populations. Following UNEAK, tags of different populations, consisting of the same reads, were used to construct network topologies. Missequenced reads, repetitive sequences, and parahomologous genes were rejected in order to obtain SNPs with high accuracy. Sequencing depth and coverage, SNP calling and genotyping were processed using BWA v. 0.7.15 and SAMtools, respectively (Li et al., 2009; Li & Durbin, 2009). In population‐genetic filters, the minor allele frequency was less than 0.10. Finally, SNPs with minimum allele frequency <0.01 (Q20 > 95%, Q30 > 90%), coverage more than four and sequencing error rate not <0.05, were retained for subsequent population genetic analysis. Conversion from Fastq files to the desired formats was carried out using PGDSpider v. 2.0.5.1 (Lischer & Excoffier, 2012).

#### 2.4.3 Population diversity, population structure and SNP phylogeograhy

The observed heterozygosity (H_O_), expected heterozygosity (H_E_), and nucletide diversity (π) were determined by VCFtool v.4.0 (Danecek et al., 2011). Population structure of *M. onukii* was inferred using three methods for the GBS datasets. The number of subgroups (K) in random mating populations was estimated by FASTStructure with a Bayesian assignment test, and the optimal K was chosen based on analyses with K from 1 to 20 replicates for cross‐validation, with clustering pattern generated by the online software Structure Plot v. 2.0. We also implemented the best model (determined by JMODELTest v. 2.1.7) to reconstruct population relationships using maximum‐likelihood (ML) with 100 bootstrap replicates in MEGA v. 7.0.14 (Kumar et al., 2016), setting *Empoasca*. (s.str.) *vitis* as the outgroup. The final tree was visualized and beautified using iTOL v. 4.0 (Letunic & Bork, 2019; http://itol.ebl.de); PCoA was performed using SNPRelate in R (Zheng et al., 2012).To confirm the results in FASTStructure, amova, *F*_ST_ and statistical significance with 1000 resampled replicates were performed in Arlequin v. 3.5 (Excoffier & Lischer, 2010).

### 2.5 Isolation by distance analysis

Isolation by distance (IBD) analyses were conducted to test whether genetic differentiation increased with geographic distance. The correlation between the linearized pairwise *F*_ST_ matrix (*F*_ST_/[1−*F*_ST_]) and the logarithm of geographic distance matrix (Log km) were estimated using a Mantel test with 999 permutations in GenALEx v. 6.5. The geographic distance between each pair of populations was estimated by Geographic Distance Matrix Generator v. 1.2.3 (Ersts, 2014).

### 2.6 Historical gene flow estimates

Given high polymorphism and mutation rates, microsatellite markers were used to estimate population size and migration parameters among clusters obtained from the results of population structure analysis. The immigration rate (*M*) per generation and the mutation‐scaled population size (*θ*) were analyzed using the Bayesian inference with full model in MIGRATE v. 3.7.2 (Beerli, 2006). The effective population size (*Ne*) of each population was calculated with *θ* = 4*Neμ* (*μ* is mutation rate, 10^−4^ per locus per generation for microsatellites) (Whittaker et al., 2003). Effective number of migrants per generation (*Nem*) was calculated as *Nem* = *θM*/4. The first run was estimated from *F*_ST_, and the other three runs were started with *θ* and *M* from the previous run. During the Bayesian search, one long chain with four independent replicates was used for each run with 10,000,000 generations after an initial burn in of 100,000 interactions. Heated chain was set to: 1.0, 1.5, 3.0, and 1000,000.

## 3 RESULTS

### 3.1 Morphological variation

**FIGURE 2:**
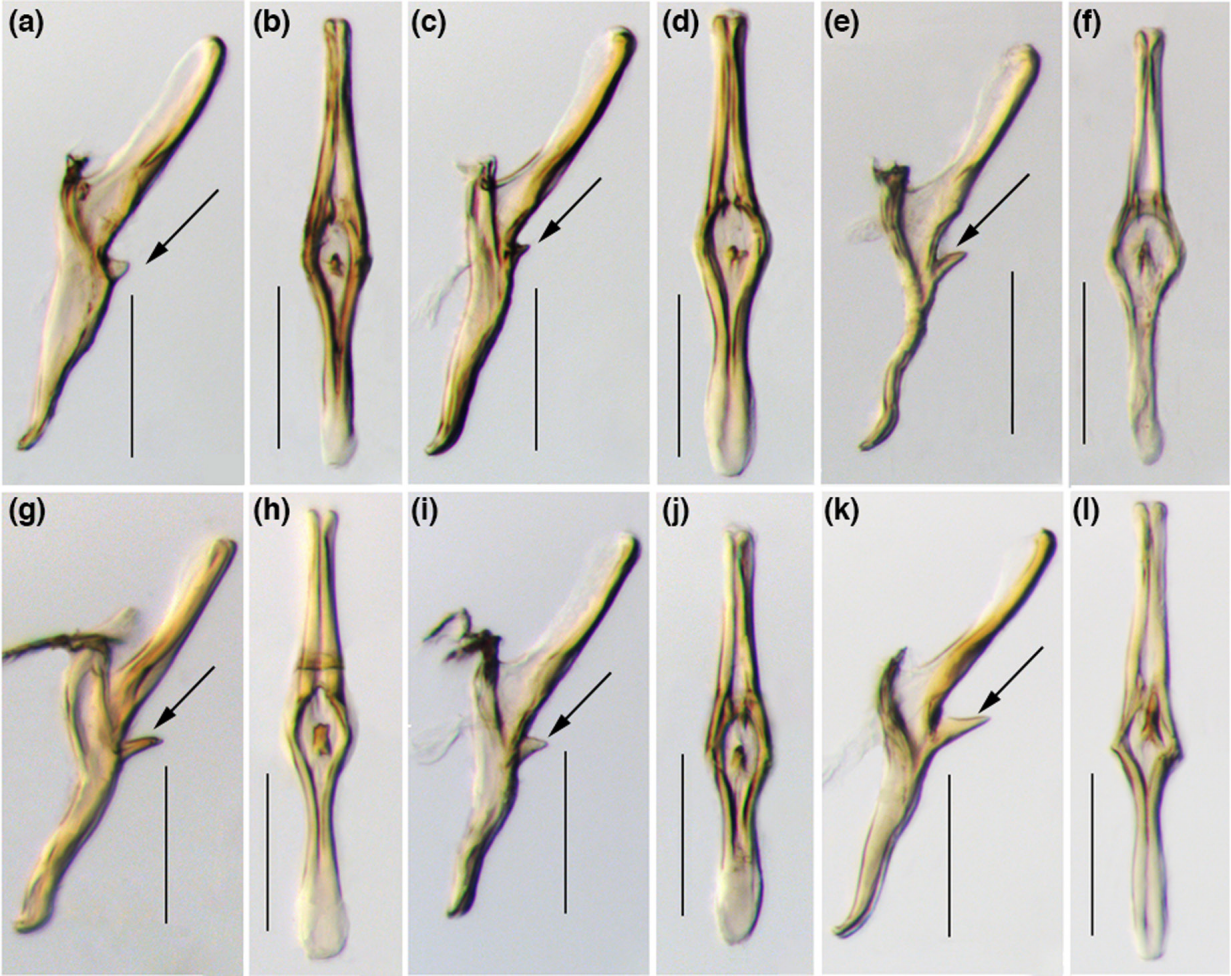
Morphological variations of aedeagus of *Matsumurasca onukii* from different tea production regions of Yunnan Province. (a) Chuxiong (CX) population, aedeagus; (b) Chuxiong (CX) population; (c) Pu′er (PE) population; (d) Pu′er (PE) population; (e) Mojiang (MJ) population; (f) Mojiang (MJ) population; (g) Lincang (LX) population; (h) Lincang (LX) population; (i) Jingdong (JD) population; (j) Jingdong (JD) population; (k) Menghai (MH) population; (l) Menghai (MH) population. (a,c,e,g,i,k) aedeagus, left lateral view; (b,d,f,h,i,j) aedeagus, ventral view. Scale bars: (a–l) = 0.1 mm. Arrows display variations in the shape of the dorsal membranous flanges and the length of ventro‐basal spiny protuberances of the aedeagal shaft.

The morphological comparisons among specimens from different populations revealed that the aedeagus of the Yunnan populations (CX, PE, MJ, MH, LX, and JD) of *M. onukii* exhibits substantial variation in the shape of the dorsal membranous flanges and the length of ventro‐basal spiny protuberances of the aedeagal shaft (Figure 2); in contrast, such variation was not observed in males from the other populations from China and populations from Japan and Vietnam. Genetic data were obtained from individuals representing these different morphological variants (spiny protuberances and no protuberances on dorsal aedeagal shaft).

### 3.2 Genetic diversity and genetic structure revealing by SSRs

The HWE test showed that 18 microsatellite markers were suitable for further genetic analysis, with low‐null allele frequencies, ranging from 0.009 to 0.081. The observed populations of *M. onukii* showed high‐genetic diversity in all tea production areas, as measured by the number of effective alleles (4.11–5.93), expected heterozygosity (0.665–0.778) and AR (7.244–10.060). Higher genetic diversity was found across *M. onukii* populations in China than in Japan (China/Japan; N_E_: 5.11/4.28, *p* = .036; AR: 8.815/7.748, *p* = .040; H_E_: 0.741/0.696, *p* = .020) (Table S1).

**FIGURE 3:**
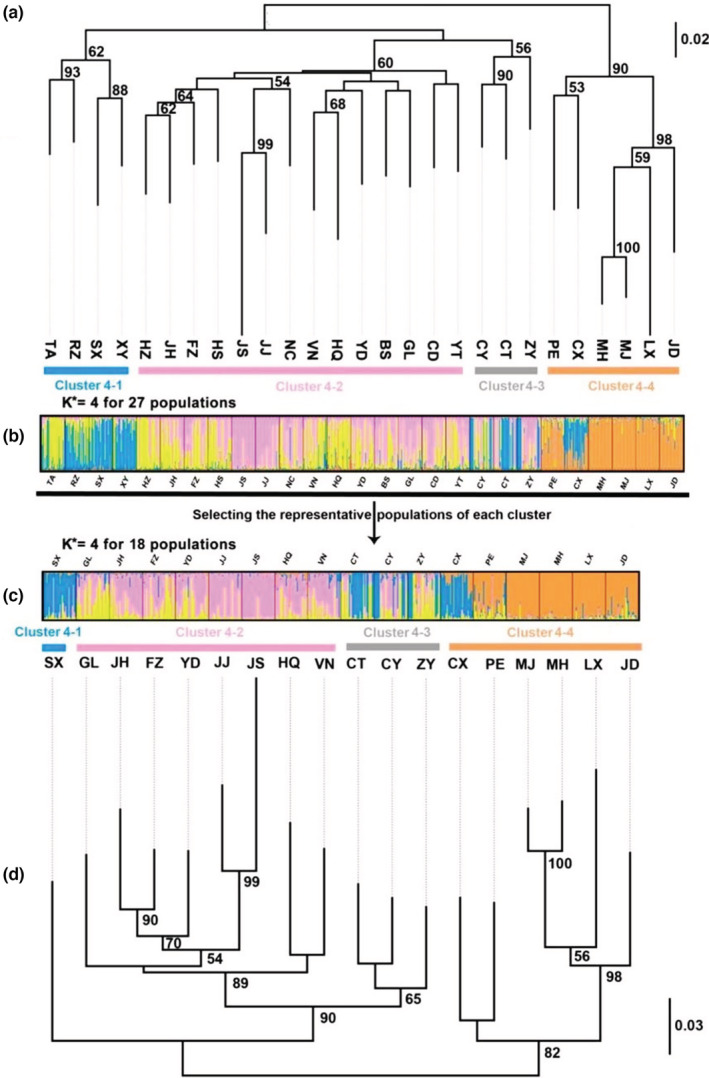
Bayesian clustering results and neighbor‐joining (NJ) tree of *Matsumurasca onukii* populations based on 18 microsatellite markers. Numbers on nodes represent bootstrap support values (values <50% not shown). The colors indicate the major clusters inferred by Bayesian clustering analysis when K = 4, except in Cluster 4–3 mixed genetic components are indicated by gray. Asterisk represent the optimal K = 4. (a,d): NJ tree for 27 populations and 18 populations; (b,c): Bayesian clustering results for 27 populations and 18 populations.

Preliminary depictions of genetic structure, as revealed by Structure v. 2.3.4, correspond with branches on the NJ tree (Figure 3a,b). Although ΔK had peaks in K = 2 and K = 4, L (K) was relatively constant at K = 4. Furthermore, individuals were strongly assigned to K = 4. East Asian populations of *M. onukii* formed four main genetic clusters: Cluster 4–1 includes four populations from Central and East China, viz. Xinyang (XY), Rizhao (RZ), Taian (TA), and Hanzhong (SX) populations; Cluster 4–2 consists of 14 populations from South China, Japan, and Vietnam, viz. Jinhua (JH), Hangzhou (HZ), Huangshan (HS), Nanchang (NC), Yichun (YT), Chengde (CD), Yingde (YD), Guilin (GL), Baise (BS), Fuzhou (FZ), Hainan (HQ), Kagoshima (JJ), Shizuoka (JS), and Vinh Phuc (VN) populations; Cluster 4–3 includes three populations from Southwest China, viz. Leshan (CT), Chongqing (CY), and Zunyi (ZY) populations; Cluster 4–4 includes six populations from Southwest China, viz. Chuxiong (CX), Pu′er (PE), Mojiang (MJ), Menghai (MH), Lincang (LX), and Jingdong (JD) populations. The six populations of *M. onukii* from the Southwest tea production area in China (CX, JD, MH, PE, LX, and MJ) were also clustered (Figure S1a,b) in the principal coordinate analysis. The amova revealed that most of the variance was due to variation within populations (93.51%, FST = 0.065; *p* < .0001); although a significant fraction of the total variation was also due to variation between clusters (2.78%, FCT = 0.028; *p* < .0001) and between populations (3.71%, FSC = 0.038; *p* < .0001) (Table 1). Low‐level genetic differentiation was detected between populations in Cluster 4–1, Cluster 4–2 and Cluster 4–3, whereas there were moderate to high levels of genetic differentiation between Yunnan (Southwest China) populations and other populations of *M. onukii* throughout the tea plantations in East Asia (*F*_ST_ > 0.050) (Figure S2).

**TABLE 1:** Analysis of molecular variance (amova) of 18 *Matsumurasca onukii* populations based on the microsatellite markers

Cluster	Source of variation	*df*	Sum of squares	Variance components	Percentage of variation (%)	Fixation indices (*p* < .0001)
K = 4	Among clusters	3	201.039	0.19071 Va	2.78	FCT = 0.028
	Among populations within clusters	14	303.325	0.25423 Vb	3.71	FSC = 0.038
	Within population	1062	6809.550	6.41201 Vc	93.51	FST = 0.065
	Total	1079	7313.914	6.85695		

### 3.3 Genetic diversity revealed by SNPs

Based on the genetic structure and genetic differentiation analyses of 27 populations of *M. onukii* in East Asia using microsatellite data, 18 populations were chosen for GBS analyses. After quality control, a final GBS dataset of 1633 SNPs representing 180 genotypes of *M. onukii* was obtained for further genetic analysis. On an average, the sequence depth of populations ranged from 5.85 to 10.84. The observed heterozygosity (range 0.231–0.364), the expected heterozygosity (range 0.210–0.291) and nucleotide diversity (range 0.198–0.275) indicated that higher genetic diversity is present in populations of *M. onukii* from the Southwest tea production areas in China (H_O_: *t* test: *t* = −2.665, *df* = 13, *p* = .019; H_E_: *t* test: *t* = −2.671, *df* = 13, *p* = .040). Among all 18 populations, the genetic diversity of populations of *M. onukii* in China was higher than those in Japan (China/Japan; π: *t* test: *t* = 3.702, *df* = 15, *p* = .002) and Vietnam (H_O_: *t* test: *t* = 5.560, *df* = 15, *p* = .000; H_E_: *t* test: *t* = 5.473, *df* = 15, *p* = .000; π: *t* test: *t* = 3.141, *df* = 15, *p* = .007) (Table S1).

### 3.4 Population structure and SNP phylogeography

**FIGURE 4:**
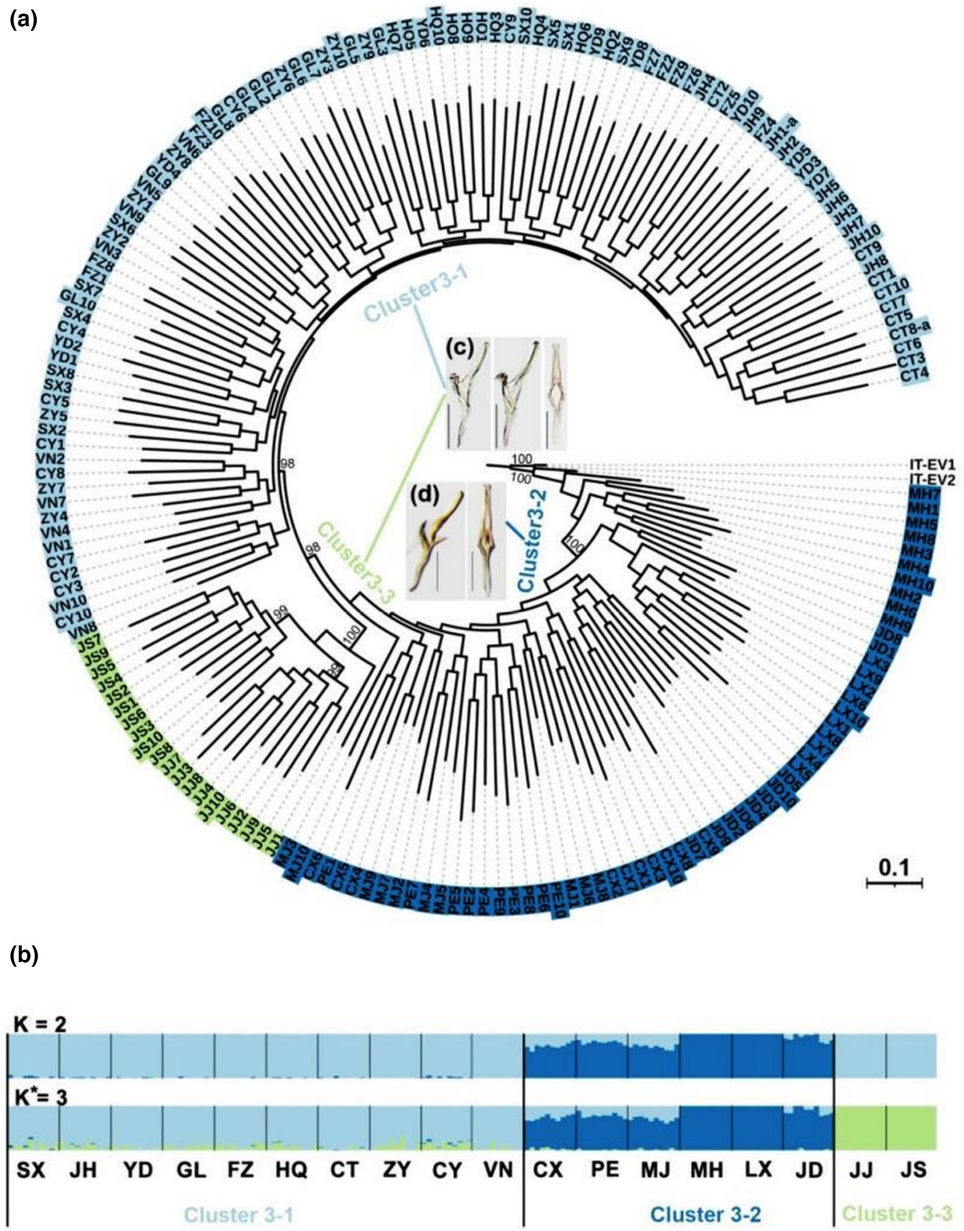
Maxmum‐likelihood (ML) tree (a) and Bayesian clustering result (b) for 180 samples of *Matsumurasca onukii* based on SNPs. Numbers on nodes represent bootstrap support values (values <50 not shown). The three clusters yielded by Bayesian clustering analysis are shown. Asterisk represent the optimal K = 3. (c) Populations in Cluster 3–1 and Cluster 3–3, aedeagus with no protuberances on left lateral view and ventral view; (d) populations in Cluster 3–2, aedeagus with spiny protuberances on left lateral view and ventral view.

The PCoA and ML analyses yielded three genetic clusters (Figure S3; Figure 4a): Cluster 3–1 contains all of Clusters 4–1 and 4–3 and part of Cluster 4–2 from the microsatellite‐based analysis: Hanzhong (SX), Jinhua (JH), Yingde (YD), Guilin (GL), Fuzhou (FZ), Hainan (HQ), Leshan (CT), Chongqing (CY), Zunyi (ZY), and Vinh Phuc (VN) populations. Cluster 3–2 is identical to Cluster 4–4 from the microsatellite‐based analysis and consists of Chuxiong (CX), Pu′er (PE), Mojiang (MJ), Menghai (MH), Lincang (LX), and Jingdong (JD) populations. Cluster 3–3 contains only the two Japanese populations: Kagoshima (JJ), Shizuoka (JS). Japanese populations grouped with Cluster 4–2 comprising populations from central China and Vietnam in the microsatellite‐based analysis although the former grouped together on a single, relatively long branch in the NJ tree. The optimal K for the SNP data inferred from FASTStructure ranged from 1 to 18 (Figure 4b), and the lowest cross‐validation error (CV) was obtained for K = 3. This clustering condition, supported by amova, revealed that populations were genetically more differentiated across the three clusters (13.38%, FCT = 0.134; *p* < .0001) than between populations (4.32%, FCT = 0.050; *p* < .0001) (Figure 4; Table 2). Pairwise *F*_ST_ ranged from 0.00039 to 0.362 for SNPs. Both datasets indicate that six populations of *M. onukii* from the Southwest tea production area in China are significantly differentiated from populations in all other sites, particularly populations in Japan (Figure S4).

**TABLE 2:** The amova of 18 *Matsumurasca onukii* populations based on reduced‐representation genome sequencing

Cluster	Source of variation	*df*	Sum of squares	Variance components	Percentage of variation (%)	Fixation indices (*p* < .0001)
K = 3	Among clusters	2	4312.070	35.64395 Va	13.38	FCT = 0.134
	Among populations within clusters	15	5013.497	11.49930 Vb	4.32	FSC = 0.050
	Within population	162	35516.900	219.24012 Vc	81.75	FST = 0.177
	Total	179	44842.467	266.38337		

### 3.5 Isolation by distance analysis

The traditional Mantel test identified significant geographic differentiation among 18 populations in both marker datasets (SNPs: *R*_*XY*_ = .461, *p* < .001; SSR: *R*_*XY*_ = .426, *p* < .000) (Figure S5). The IBD signal within populations from Southwest China was considerable when based on SNPs (*R*_*XY*_ = .647, *p* = .002), but not significant based on microsatellite markers (*R*_*XY*_ = .319, *p* = .061). IBD tests revealed no significant geographic differentiation in genetic clusters (Cluster 3–1: *R*_*XY*_ = .220, *p* = .196) with weak genetic differentiation and small distribution (Figure S6).

### 3.6 Historical gene flow estimates

**FIGURE 5:**
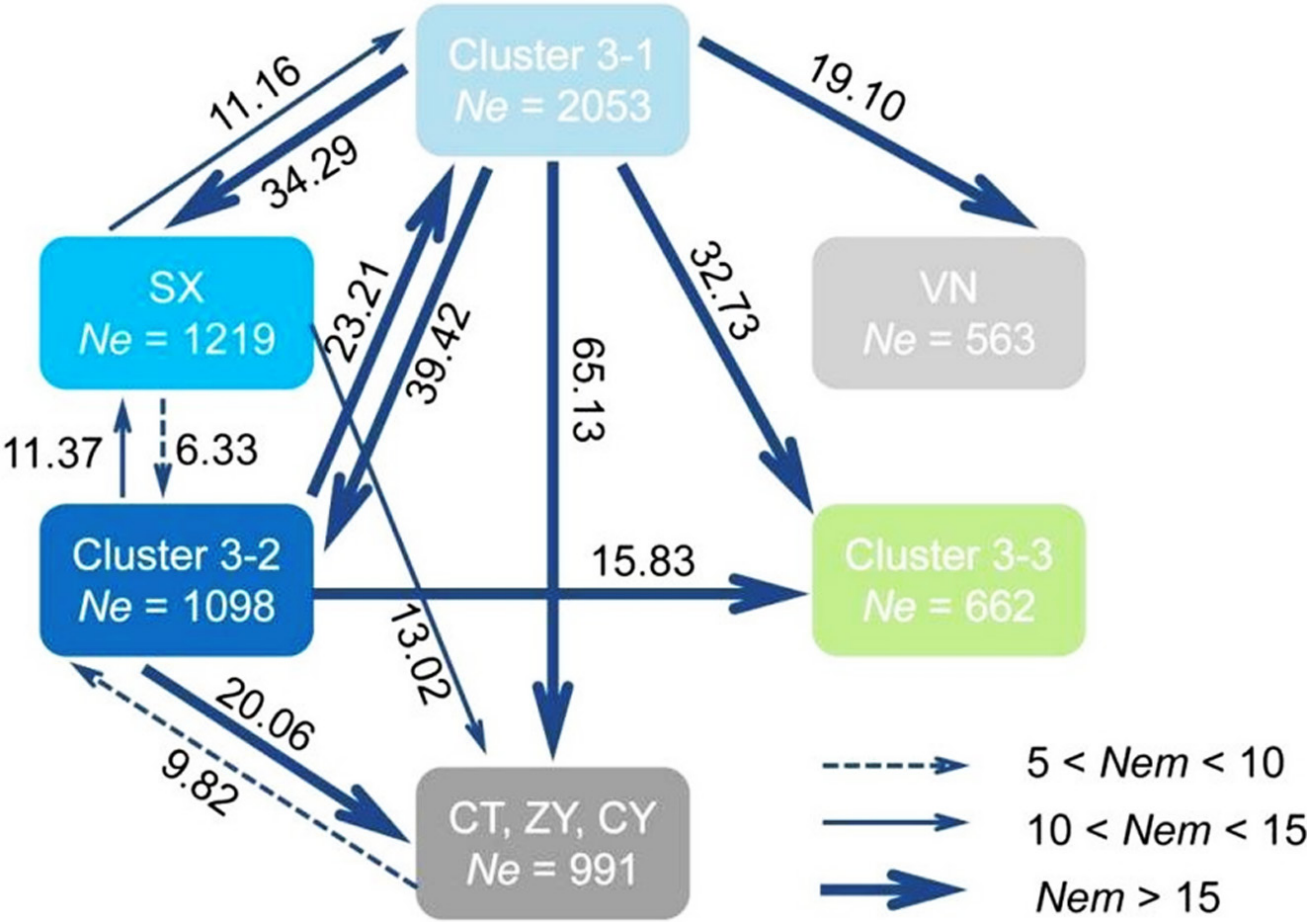
Maximum likelihood estimates for gene flow among clusters using SSRs. Cluster 3–1 excepted for Hanzhong (SX), Vinh Phuc (VN), Leshan (CT), Chongqing (CY), and Zunyi (ZY) populations (included Jinhua (JH), Yingde (YD), Guilin (GL), Fuzhou (FZ), and Hainan (HQ) populations), Cluster 3–2 (Yunnan populations included Chuxiong (CX), Pu′er (PE), Mojiang (MJ), Menghai (MH), Lincang (LX), and Jingdong (JD) populations), Cluster 3–3 (included Kagoshima (JJ) and Shizuoka (JS) populations) and Hanzhong (SX), Vinh Phuc (VN) populations, and Leshan (CT)/Chongqing (CY)/Zunyi (ZY) populations. Ne inside circles is effective population size (i.e., *θ* = 4 *Neμ*, where *θ* is the population size parameter and *μ* is mutation rate) and *Nem*: Effective number of migrants per generation (i.e., *Nem = θM*/4, where *M* is scaled migration rate per generation) for *Matsumurasca onukii*. Arrows indicate the gene flow direction among clusters. *Nem* < 5 and no significant asymmetrical effective migrants not shown.

Given the differences in results of genetic structure analyses based on SNPs versus microsatellites, estimates of gene flow among population clusters identified by both analyses were made. In order to clarify the relationship between populations in China and Vietnam, the VN population was also separated for gene flow estimates. Effective cluster size calculated with population size parameter *θ*, ranged from 563 to 2053. Frequent exchange of migrants per generation was inferred to occur among clusters (Figure 5). High levels of bidirectional migration were inferred between Clusters 3–1 and 3–2 (*Nem* > 20). Asymmetric migration was found among clusters in China and between clusters in China, Japan, and Vietnam, with effective number of migrants per generation (*Nem*) from Cluster 3–1 and Cluster 3–2 to SX (34.29 and 11.37) higher than numbers of migrants in the opposite direction (11.16 and 6.33). CT/CY/ZY populations displayed asymmetric migration from Cluster 3–1 (65.13), Cluster 3–2 (20.06) and SX populations (13.02); *Nem* from Cluster 3–1 and Cluster 3–2 to Cluster 3–3 were 32.73 and 15.83; and immigrants to the VN population were mainly from Cluster 3–1 (19.10). When SX population or CT/CY/ZY populations were included in Cluster 3–1 based on the results by SNP dataset, obvious asymmetric migration was also detected (Figure S7).

## 4 DISCUSSION

Accurate assessment of genetic structure is a major aim of pest molecular ecology, as it can reveal patterns of association among populations and contribute toward ecology‐based management methods. In this study, we compared assessments of population structure in *M. onukii* based on two different sets of markers, SNPs and SSRs. Our SNP dataset confirmed some genetic structural patterns in *M. onukii* revealed by prior studies based on microsatellites (Zhang et al., 2019) but also revealed some differences in genetic diversity, relatedness, population structure and geographic differentiation.

### 4.1 Hierarchical pattern of *M. onukii* genetic structure based on microsatellites versus SNPs

As in the prior analysis based on microsatellites, analysis of SNPs showed that populations in the Yunnan tea production area of China are genetically differentiated from the other populations (Zhang et al., 2016, 2019). Nevertheless, the present analysis based on SNPs revealed an additional genetic cluster consisting only of the two Japanese populations (Cluster 3–3 in Figure 4a) which was not recognized by Zhang et al. (2016, 2019), and also uncovered finer‐scale geographic differentiation. The findings are consistent with other studies on various species, such as *Laodelphax striatellus*, *Plasmodium vivax*, *Carassius carassius*, and so on (Fola et al., 2020; Jeffries et al., 2016; Liu et al., 2019), where SNPs outperformed microsatellite markers in accuracy and clarifying finer population structure.

Our results suggest that microsatellites and SNPs may capture somewhat different aspects of population structure in *M. onukii* although the reasons for these differences remain to be explored. Microsatellite markers tend to exhibit higher levels of homoplasy, null alleles, and usually reveal a large number of alleles at a locus, resulting in underestimated genetic differentiation between subpopulations with high‐genetic distance (e.g., Chinese populations and Japanese populations) and identify population structure between populations with similar levels of genetic diversity, which is consistent to many prior studies (Campos et al., 2017; Fola et al., 2020). In contrast, SNPs have a fixed number of allelic states (Elshire et al., 2011), and SNPs with slower mutation rates may also facilitate inferring higher level of genetic differentiation and provide additional discrimination among genetic clusters (Coates et al., 2009; Morin et al., 2004). This may explain why the two Japanese populations formed their own separate cluster in the analysis based on SNPs (Cluster 3–3 in Figure 4a) while these populations grouped within a large cluster including populations from central China and Vietnam in the microsatellite‐based analysis (Cluster 4–2 in Figure 3d). Similarly, the CT, CY, ZY, and SX populations were clustered with Cluster 4–1 and Cluster 4–3 based on microsatellite data (Figure 3c,d), while they were clustered with JH, YD, GL, FZ, HQ, and VN populations in Cluster 3–1 by the SNPs data (Figure 4a,b).

Our results imply that different mutation rates of the two types of markers might reflect different stages of population evolutionary history, which can lead to differences in estimated population structure. Microsatellite markers, with higher mutation rates, tend to provide insights mostly on recent divergences by compared to SNPs (Sun et al., 2009). Transfer of famous tea varieties among regions has a long history in China (Ruan, 2019) and presumably resulted in occasional transport of tea seedlings with eggs and adults of *M. onukii* over long distances between tea production areas. Frequent migration or human‐mediated exchange between and within populations may have yielded similar genetic structure of *M. onukii* populations in most of tea production areas of China, except for the Southwest (particularly in Yunnan Province). The “introduction from south to north” mainly occurred in recent decades, during which major tea planting areas were established in North China (An et al., 2014). Southwest China (included Yungui Plateau and Sichuan–Chongqing region) has been confirmed as the original area of tea production (Chen, 2009; Chen et al., 2019; Yao et al., 2008). At present, transfer of fresh tea leaves among some famous tea planting areas is occurring frequently, especially in the Southwest tea areas bordering on the other tea areas (e.g., Sichuan, Chongqing, Guizhou Provinces). Differences observed among *M. onukii* populations may have resulted from occasional population bottlenecks and selection based on adaptation to local environmental conditions as *M. onukii* populations migrate along with their host plants, accounting for similar genetic diversity and structure in Cluster 4–1 and higher genetic diversity and hybrid genetic structure in Cluster 4–3 shown by microsatellites, whereas SNPs detected different scale geographic population structure that may be a result of reduced gene flow due to geographic barriers present for a long time (e.g., between Southwest China and the remaining populations and between China and Japan) (Figure 5).

### 4.2 Correlations between morphological and geographic distance and genetic variation

Although we detected no significant genetic substructure in most Chinese tea areas, our analyses of two types of markers revealed special genetic structure and high‐genetic differentiation in *M. onukii* populations from the Southwest tea areas, especially among the populations from Yunnan tea plantations that also exhibit morphological variation in the aedeagus. Such correlation between genetic and morphological variation contrasts with a previous study of *Asymmetrasca decedens*, in which no genetic differentiation was found between individuals of two morphotypes based on differences in the shape of the aedeagus, (Loukas & Drosopoulos, 1992). The CX, PE, MJ, JD, LX, and MH populations of *M. onukii* were collected from cultivated and wild Yunnan big leaf tea in relatively isolated environments, while the other populations of China and Vietnam were from fewer varieties planted over larger areas.

Our analysis using two markers also revealed that the Kagoshima (JJ) and Shizuoka (JS) populations from Japan and the Vinh Phuc (VN) population from Vietnam are closely related to *M. onukii* populations in China. Not surprisingly, the VN population is more similar to the Chinese populations than are the two populations from Japan (Figures 3b and 4a). Japanese populations also showed relatively high levels of genetic differentiation and lower genetic diversity compared with the Chinese populations, indicating that gene flow between Chinese and Japanese populations is very low. Genetic drift and natural selection on the limited genetic resources of *M. onukii* in Japan may have yielded low‐genetic diversity and high differentiation compared with the mainland populations (Kaundun et al., 2000; Wachira et al., 2001; Yao et al., 2008). In contrast, high‐genetic diversity and less genetic variation were revealed in the Vietnamese population as expected, given the absence of significant geographical barriers, similar host resources, and frequency of gene exchange between VN population and JH, YD, GL, FZ, HQ, CT, CY, and ZY populations (Figures 3b and 4a; Figures S2 and S4). As mentioned earlier, genetic structure of *M. onukii* may be affected by variation in tea germplasm resources and habitat among tea plantations.

### 4.3 Genetic connectivity and population dynamics

Analyses of population structure and phylogeography based on the SNPs reveal significant genetic differentiation between *M. onukii* populations in Yunnan (Cluster 3–2), Japan (Cluster 3–3) and the other tea plantations of China and Vietnam. Significant asymmetrical historical migration from Cluster 3–1 and Cluster 3–2 to Cluster 3–3 is supported by non‐overlapping 95% confidence intervals (Figure 5; Figure S7). Previous studies based on mitochondrial markers indicated that few private haplotypes are present in the Yunnan populations (Zhou et al., 2014; Zhu et al., 2019), and the putative primitive haplotype was also present in populations from Yunnan and neighboring Sichuan, Guizhou, and Chongqing Provinces (Chen et al., 2015; Li et al., 2013). According to genetic diversity, genetic structure characteristics, historical gene flow of *M. onukii* populations revealed by this study and historical expansion of tea cultivation (Takeo et al., 1992), we hypothesize that the tea green leafhopper in China spread from Yunnan or the southwest populations to the other tea areas in Jiangnan, South China and, ultimately, to Japan with domestication and cultivation (the historical sources), and then returned back to the origin by recent events (such as fresh tea processing and tea germplasm exchange) forming a new diffusion source pattern. As a result, the leafhopper was most likely introduced to Japan tea plantations through these two diffusion sources from China (Figure 5; Figure S7). We found no evidence of frequent, long‐distance directional dispersal, as has been observed in some other species of Empoascini (e.g., the potato leafhopper, *Empoasca fabae*) in *M. onukii*. Instead, our results suggest that natural or human‐assisted dispersal occurs among major tea growing areas of China but not frequently enough to prevent maintenance of population‐level differences between major tea areas and high levels of genetic variability within some populations.

Additional genetic substructure in *M. onukii* populations throughout East Asia was further elucidated in this study by SNPs, and high levels of genetic differentiation were observed between populations in different clusters. But our analyses failed to reveal fine‐scale dispersal routes between populations. Incorporation of additional SNPs into future studies by more efficient genotyping with the *M. onukii* reference genome (ASM1883171v1) in Genome Warehouse may allow for finer‐scale analysis of gene flow among populations (Malinsky et al., 2021; Pickrell & Pritchard, 2012).

## 5 CONCLUSION

Recent advances in sequencing technologies can provide genomic data for accurately identifying patterns of genetic structure with effective samples. The accurate genetic structure will be obtained when the gene markers and samples are chosen reasonably. In this study, representative populations of *M. onukii* were chosen by the genetic structure based on microsatellite markers, which GBS‐derived SNPs and microsatellite markers all captured multiscale patterns of genetic structure. Three clusters were discovered in East Asia by SNPs, performed better than the microsatellite markers at fine spatial scales. General migration route showed that there are two diffusion sources including Yunnan and the other tea plantations of China, respectively, from where introduced to Japan tea plantations. Our findings indicate that the host plants (e.g., special tea germplasm resources) and nature geographical condition (e.g., geographical barriers) may be significant involved to the genetic variation of *M. onukii* between different Clusters.

Therefore, to illuminate fine‐scale dispersal routes of *M. onukii*, further study should encompass more SNPs and additional samples from various host plants within *Camellia* spp. at fine scales (such as within the southwest tea planting area). Higher‐resolution study of gene flow and its relation to geography/host associations will help elucidate the microevolution of adaptability in *M. onukii* populations in the East Asia.

## AUTHOR CONTRIBUTIONS

**Li Zhang:** Conceptualization (equal); formal analysis (lead); investigation (lead); resources (equal); software (equal); writing – original draft (lead); writing – review and editing (equal). **Christopher H. Dietrich:** Conceptualization (supporting); writing – original draft (supporting); writing – review and editing (equal). **Ye Xu:** Investigation (supporting); writing – original draft (supporting); writing – review and editing (equal). **Zhaofu Yang:** Software (equal); writing – review and editing (equal). **Maohua Chen:** Conceptualization (supporting); methodology (lead); writing – review and editing (equal). **Thái H. Pham:** Funding acquisition (supporting); resources (supporting); writing – review and editing (supporting). **Cuong C. V. Le:** Resources (supporting); writing – review and editing (supporting). **Li Qiao:** Resources (supporting); writing – review and editing (supporting). **Masaya Matsumura:** Resources (supporting); writing – review and editing (supporting). **Daozheng Qin:** Conceptualization (equal); funding acquisition (lead); investigation (supporting); resources (lead); writing – original draft (supporting); writing – review and editing (equal).

## CONFLICT OF INTEREST

The authors declare that there are no conflicts of interest.

## BENEFIT‐SHARING STATEMENT

Benefits from this research accrue from the sharing of our data and results on public databases as described earlier. No specific permits were required for this study. Tea green leafhopper, *M. onukii*, is an agricultural pest, not an endangered or protected species. All the samples were collected in open tea plantations and not from any national parks or protected areas.

## Supporting information


Appendix S1

## Data Availability

Individual genotype data of *M. onukii* are available on Dryad (https://datadryad.org/stash/share/muyHpreBGuAO‐Kanv0KS5L6n7i4p‐wgJ6KfgZ6qqFBM). The raw Illumina reads are archived at the NCBI Sequence Read Archive (SRA) under BioProject ID PRJNA799739.
