# Supplementary material for: Unraveling the hierarchical genetic structure of tea green leafhopper, Matsumurasca onukii, in East Asia based on SSRs and SNPs
Source: Ecol Evol. 2022 Oct 1;12(10):e9377. doi: 10.1002/ece3.9377 (PMC9526121; doi:10.1002/ece3.9377)
Supplement: Supplementary file 1 — Appendix S1 [file ECE3-12-e9377-s001.docx]

**SUPPORTING MATERIALS**

**Table S1** Collecting information and genetic diversity of *Matsumurasca onukii* populations. Geographic coordinates, number of samples collected (N), effective number of alleles (N_E_), allelic richness (AR), observed heterozygosity (H_O_) and expected heterozygosity (H_E_) based on SSRs from 27 geographical populations of *M.* *onukii*; observed heterozygosity (H_O_), expected heterozygosity (H_E_) and nucleotide diversity (π) based on SNPs from 18 geographical populations of *M.* *onukii*

| Code | Collecting locality | Latitude /  Longitude | N | SSRs | | | |  | SNPs | | |
| --- | --- | --- | --- | --- | --- | --- | --- | --- | --- | --- | --- |
|  |  |  |  | N_E_ | AR | H_O_ | H_E_ |  | H_O_ | H_E_ | π |
| **SX** | Hanzhong, Shaanxi | 32.98°/  107.77° | 30 ♂ | 5.93 | 9.415 | 0.699 | 0.780 |  | 0.263 | 0.229 | 0.260 |
| **JH** | Jinhua,  Zhejiang | 28.89°/  119.82° | 30 ♂ | 5.59 | 9.430 | 0.670 | 0.745 |  | 0.281 | 0.238 | 0.267 |
| **YD** | Yingde, Guangdong | 24.30°/  113.40° | 30 ♂ | 5.49 | 9.185 | 0.656 | 0.733 |  | 0.268 | 0.229 | 0.266 |
| **GL** | Guilin,  Guangxi | 25.28°/  110.34° | 30 ♂ | 5.14 | 9.063 | 0.680 | 0.740 |  | 0.263 | 0.230 | 0.257 |
| **FZ** | Fuzhou,  Fujian | 26.08°/  119.24° | 30 ♂ | 5.65 | 9.384 | 0.707 | 0.745 |  | 0.269 | 0.230 | 0.262 |
| **HQ** | Qiongzhong, Hainan | 19.05°/  109.92° | 30 ♂ | 5.03 | 8.696 | 0.701 | 0.750 |  | 0.269 | 0.233 | 0.252 |
| **CT** | Leshan,  Sichuan | 29.79°/  103.69° | 30 ♂ | 5.67 | 9.646 | 0.697 | 0.770 |  | 0.282 | 0.237 | 0.251 |
| **ZY** | Zunyi,  Guizhou | 27.77°/  107.48° | 30 ♂ | 5.48 | 9.062 | 0.615 | 0.738 |  | 0.255 | 0.225 | 0.255 |
| **CY** | Yongchuan, Chongqing | 29.40°/  105.92° | 30 ♂ | 5.89 | 10.060 | 0.668 | 0.788 |  | 0.238 | 0.216 | 0.248 |
| **CX** | Chunxiong, Yunnan | 24.57°/  101.81° | 30 ♂ | 4.96 | 8.936 | 0.700 | 0.754 |  | 0.293 | 0.249 | 0.275 |
| **PE** | SimaoDistrict,  Puer, Yunnan | 22.75°/  100.96° | 30 ♂ | 4.47 | 8.007 | 0.646 | 0.699 |  | 0.303 | 0.254 | 0.273 |
| **MJ** | Mojiang,  Puer, Yunnan | 23.10°/  101.61° | 30 ♂ | 4.11 | 7.807 | 0.633 | 0.698 |  | 0.246 | 0.224 | 0.245 |
| **MH** | Menghai, Yunnan | 21.98°/  100.43° | 30 ♂ | 4.26 | 7.647 | 0.654 | 0.713 |  | 0.325 | 0.273 | 0.216 |
| **LX** | Lincang, Yunnan | 23.85°/  100.03° | 30 ♂ | 4.26 | 7.244 | 0.665 | 0.717 |  | 0.364 | 0.291 | 0.214 |
| **JD** | Jiangdong,  Puer, Yunnan | 24.45°/  100.83° | 30 ♂ | 4.52 | 8.390 | 0.703 | 0.735 |  | 0.263 | 0.229 | 0.260 |
| **JJ** | Kagoshima, Japan | 31.60°/  130.56° | 30 ♂ | 4.18 | 7.955 | 0.632 | 0.665 |  | 0.281 | 0.238 | 0.267 |
| **JS** | Shizuoka,  Japan | 34.84°/  138.18° | 30 ♂ | 4.38 | 7.541 | 0.663 | 0.726 |  | 0.268 | 0.229 | 0.266 |
| **VN** | Vinh Phuc, Vietnam | 21.38°/  105.71° | 30 ♂ | 5.33 | 9.625 | 0.696 | 0.767 |  | 0.263 | 0.230 | 0.257 |
| XY | Xinyang,  Henan | 32.09°/  114.06° | 30 ♂ | 5.35 | 8.965 | 0.704 | 0.767 |  | - | - | - |
| RZ | Rizhao, Shandong | 35.29°/  119.26° | 30 ♂ | 5.00 | 8.665 | 0.737 | 0.760 |  | - | - | - |
| TA | Taian,  Shandong | 36.17°/  117.24° | 30 ♂ | 4.83 | 8.596 | 0.664 | 0.738 |  | - | - | - |
| HZ | Hangzhou, Zhejiang | 30.21°/  120.09° | 30 ♂ | 5.46 | 9.334 | 0.700 | 0.749 |  | - | - | - |
| HS | Huangshan, Anhui | 29.85°/  117.72° | 30 ♂ | 5.00 | 8.169 | 0.713 | 0.745 |  | - | - | - |
| NC | Nanchang, Jiangxi | 28.81°/  115.72° | 30 ♂ | 4.89 | 8.484 | 0.635 | 0.702 |  | - | - | - |
| YT | Yichun,  Jiangxi | 28.52°/  114.37° | 30 ♂ | 5.47 | 9.292 | 0.711 | 0.740 |  | - | - | - |
| BS | Baise,  Guangxi | 24.50°/  106.66° | 30 ♂ | 5.93 | 9.415 | 0.699 | 0.780 |  | - | - | - |
| CD | Changde,  Hunan | 28.64°/  111.16° | 30 ♂ | 5.59 | 9.430 | 0.670 | 0.745 |  | - | - | - |

The bold code represent that populations chosen for further analysis using SNPs.

**Table S2** Characteristics of 18 microsatellite markers analyzed in *M.* *onukii*

| Markers (GenBank accession no.) | Repeat | Primer sequence (5'–3') | Size range (bp) | Observed number of alleles |
| --- | --- | --- | --- | --- |
| *Eo*-29  (KU588268) | (TC)_6_ | F:[TAMRA]-CCAGTGAGGAAAGGAGGA  R:GGGTATGATTAGCGGTGT | 140-176 | 16 |
| *Eo*-51  (KU588269) | (CT)_11_GT(CT)_4_ | F:[FAM]-TCGCTCCACTCTACCACT  R:CTAAAACTAACAAATCCACCT | 188-246 | 28 |
| *Eo*-37  (KU588270) | (AG)_6_ | F:[TAMRA]-GTTTTGGGTATGATTAG  R:CCAGTGAGGAAAGGAG | 153-181 | 14 |
| *Eo*-54  (KU588271) | (GA)_8_ | F:[FAM]-CTGTTCGCAGTTCACATCATTC  R:GACCCGCTACGCTTACCTATT | 399-459 | 24 |
| *Eo*-1-61  (KU588272) | (CA)_4_…(CA)_4_ | F:[HEX]-CGGCATTCATTATCTC  R:CACACAACTCACTCGCT | 82-112 | 16 |
| *Eo*-1-52  (KU588273) | (AC)_12_ | F:[FAM]-GCCGTGTGTAATGGTATCC  R:GACGCCTAGCAATGTT | 195-247 | 22 |
| *Eo*-42  (KU588274) | (GA)_8_ | F:[HEX]-GGACAATGAAAATTCGAGGACGG  R:GGCAATCGGCAACAACAAAC | 111-179 | 29 |
| *Eo*-1-82  (KU588275) | (GT)_14_ | F:[FAM]-TGACAGCCATAAACACCG  R:CGTAGACCAGATGACCCTC | 264-308 | 23 |
| *Eo*-20  (KU588277) | (GA)_9_ | F:[TAMRA]-CTGTCACTTGCCAATAACTCT  R:CAACCACCTCACTCCCTCT | 127-169 | 19 |
| *Eo*-1-57  (KU588279) | (AC)_6_ | F:[HEX]-TACTCACCGCTCGTCTATC  R:CACTTTTATTTTCGGCTCT | 93-111 | 8 |
| *Eo*-83  (KU588280) | (AG)_6_ | F:[HEX]-GAGTCTGTTCGGTTTGATGT  R:CAAGCGATAAGCAAGGTAAG | 90-132 | 12 |
| *Eo*-E-12  (KU588281) | (GT)_14_ | F:[TAMRA]-GCAAGCCGTTAGCATAGT  R:AACACCGCATTTCATACA | 123-185 | 27 |
| *Eo*-70  (KU588282) | (AC)_9_C(CA)_12_ | F:[FAM]-CAGGAGCAGGACAAGAG  R:GCACATAAGCCTAAACAGAC | 179-269 | 29 |
| *Eo*-36  (KU588284) | (AG)_9_ | F:[FAM]-CCAGACAGCGAAGTGAAT  R:GGTACCCGAAGGAAGGAT | 257-323 | 22 |
| *Eo*-68  (KU588285) | (AG)_4_GG(AG)_3_ | F:[TAMRA]-TAGGGGTTCGACAGACTTG  R:GAGGTGAGGTTGGGATTTG | 146-198 | 23 |
| *Eo*-1-5  (KU588288) | (GTT)_4_ | F:[TAMRA]-CCGAAGAAGTCCAAGATAA  R:CGAGGAGGAGGCTAAAG | 121-148 | 8 |
| *Eo*-F-8  (KU588278) | (CA)_13_G(AC)_5_ | F:[HEX]-CCTTGTAATGCGATGC  R:GATGACACTGCCGAAAC | 92-138 | 23 |
| *Eo*-1-77  (KU588287) | (AC)_12_ | F:[TAMRA]-TTCCGTGTGTAATGGTATC  R:CCAATGTTGTTTGCGAC | 191-231 | 18 |


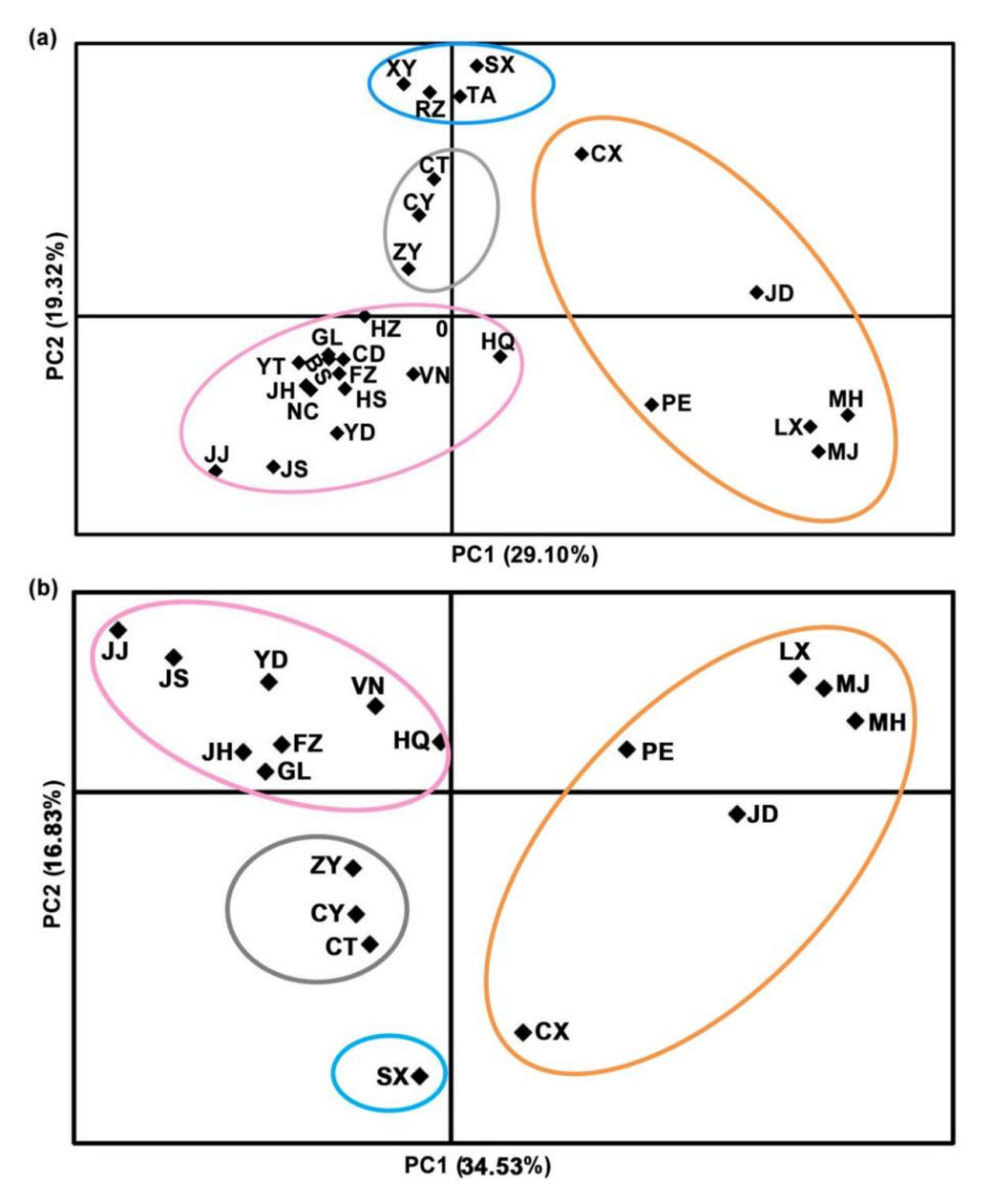


**Fig. S1** PCoA at population level generated from microsatellite markers. (a) The first two principle components for 27 populations, PC1 and PC2, account for 29.10% and 19.32% of total variance; (b) The first two principle components for 18 populations, PC1 and PC2, account for 34.53% and 16.83% of total variance. Blue and Pink are coded as in Figure 3.


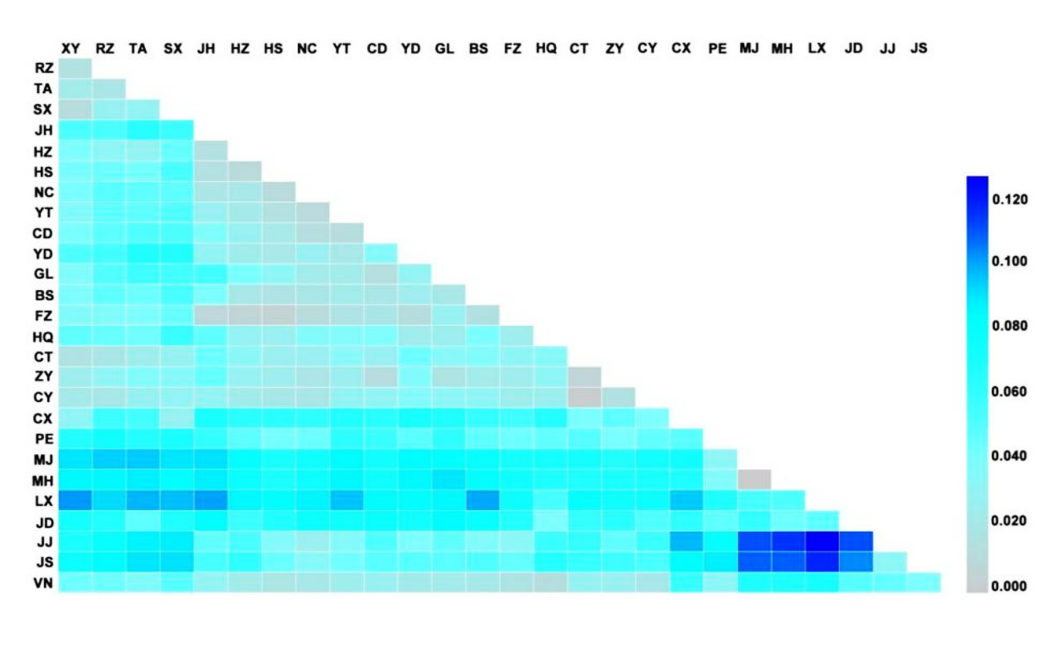


**Fig. S2** Heat map of *F*_ST_ between 27 *M.* *onukii* populations


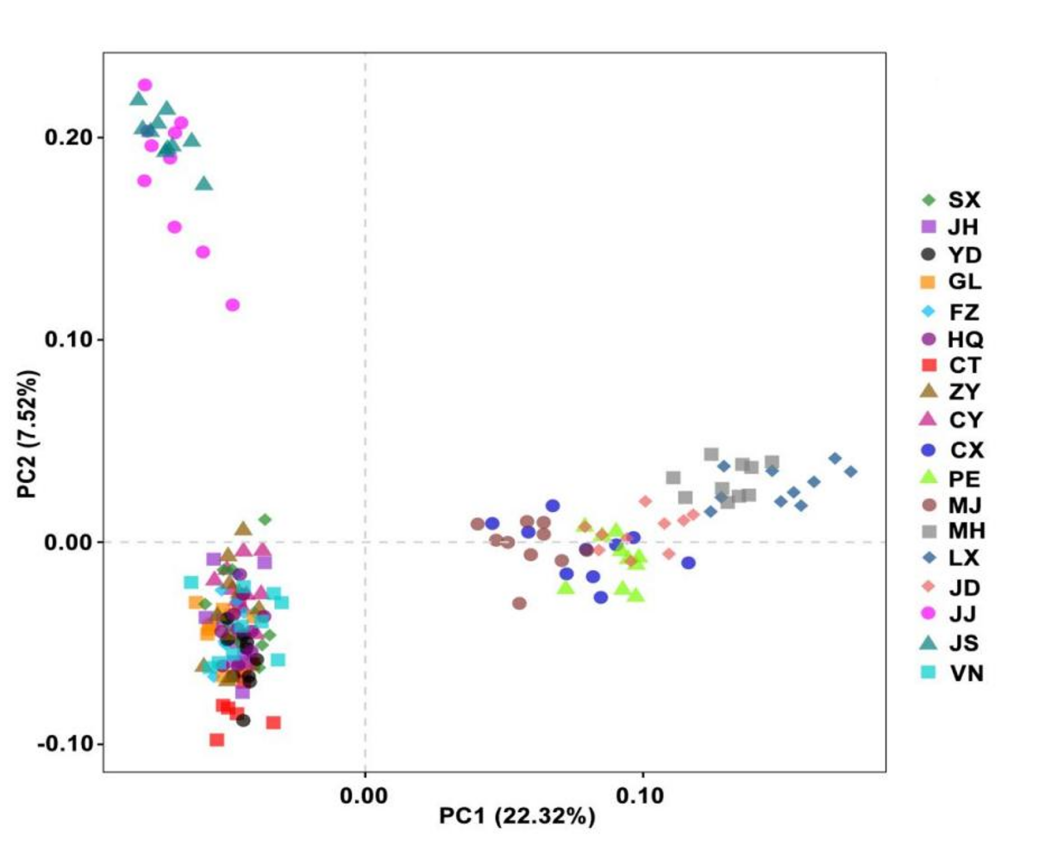


**Fig. S3** PCoA generated from reduced-representation genome sequencing in 18 *M.* *onukii* populations. The first two principle component factors, PC1 and PC2, account for 43.76% and 26.48% of total variance.

.


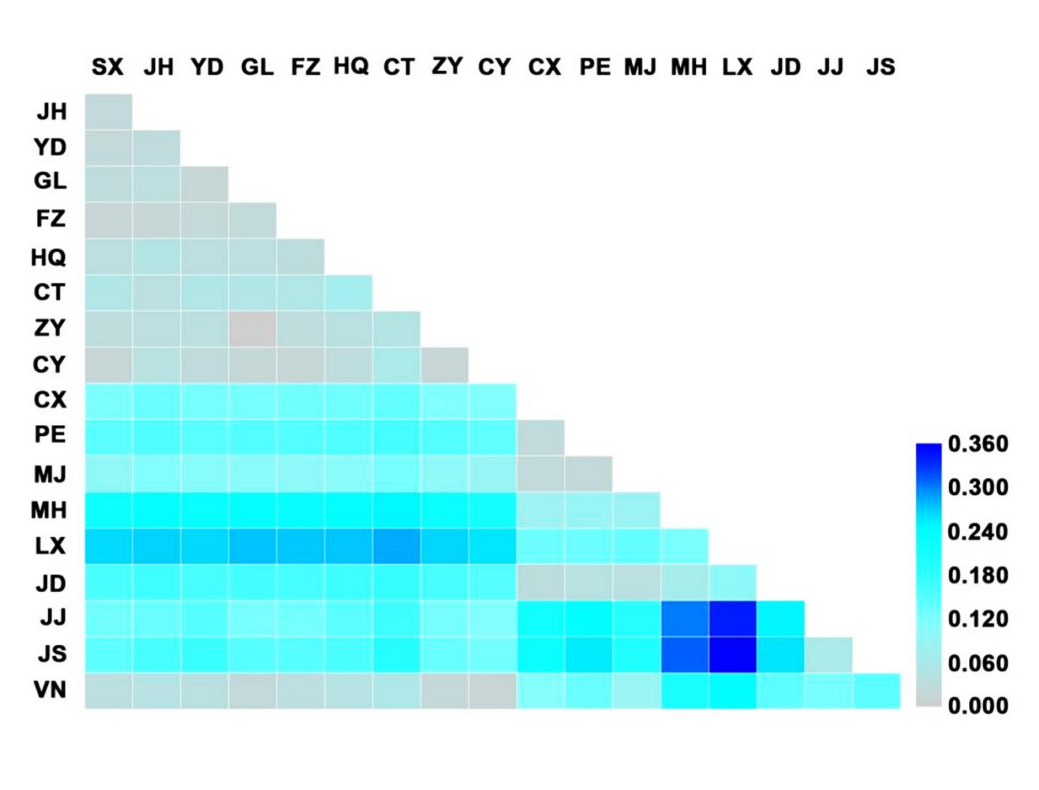


**Fig. S4** Heat map of *F*_ST_ between 18 *M.* *onukii* populations


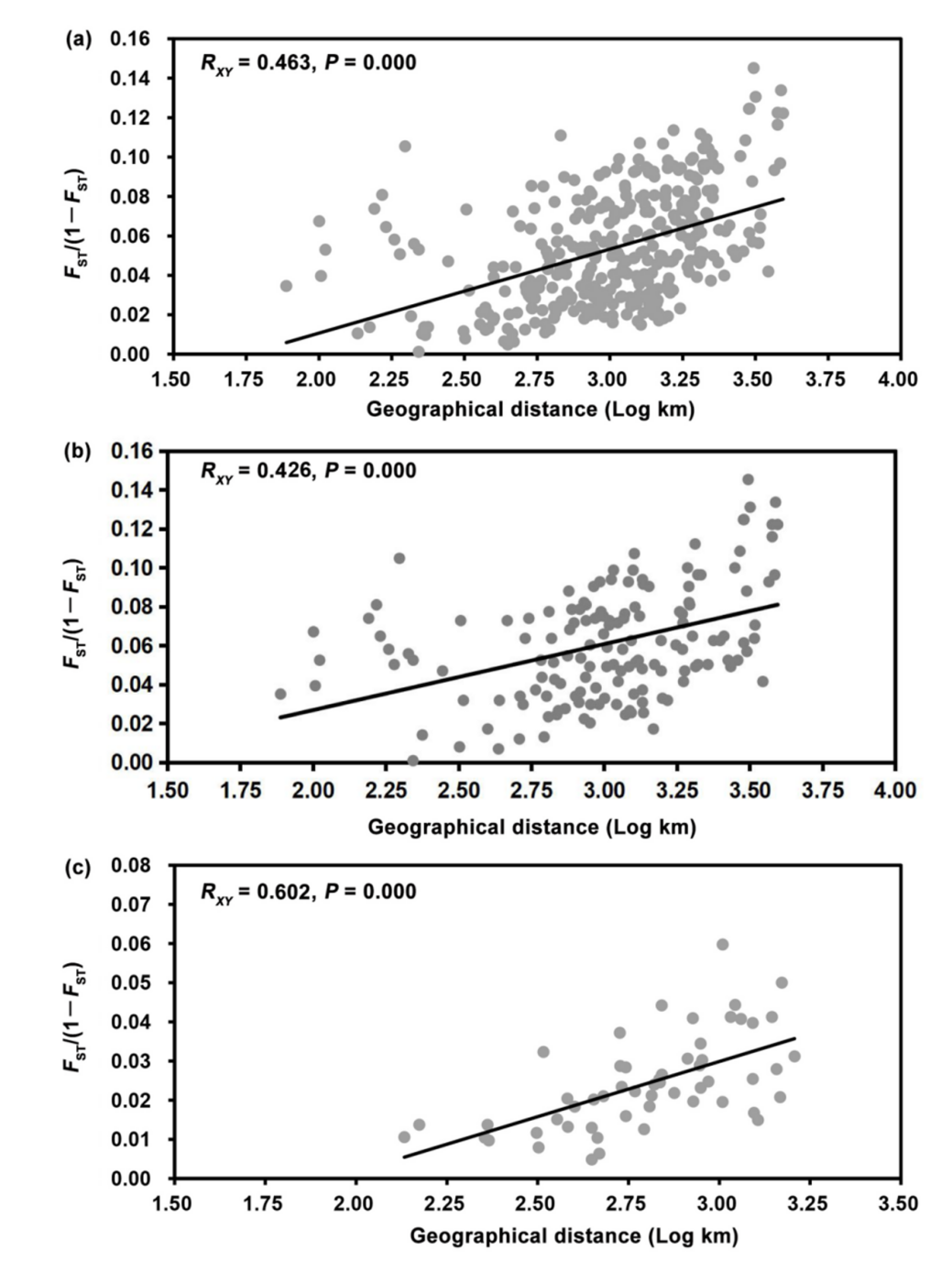


**Fig. S5** Correlation analysis between pairwise *M.* *onukii* population estimates of linearized *F*_ST_/(1－*F*_ST_) and the logarithms of geographic distance based on 18 microsatellite markers. (a) 27 populations; (b) 18 population in China; (c) Cluster 4-2. Cluster 4-2 inferred by Bayesian clustering analysis when K = 4.


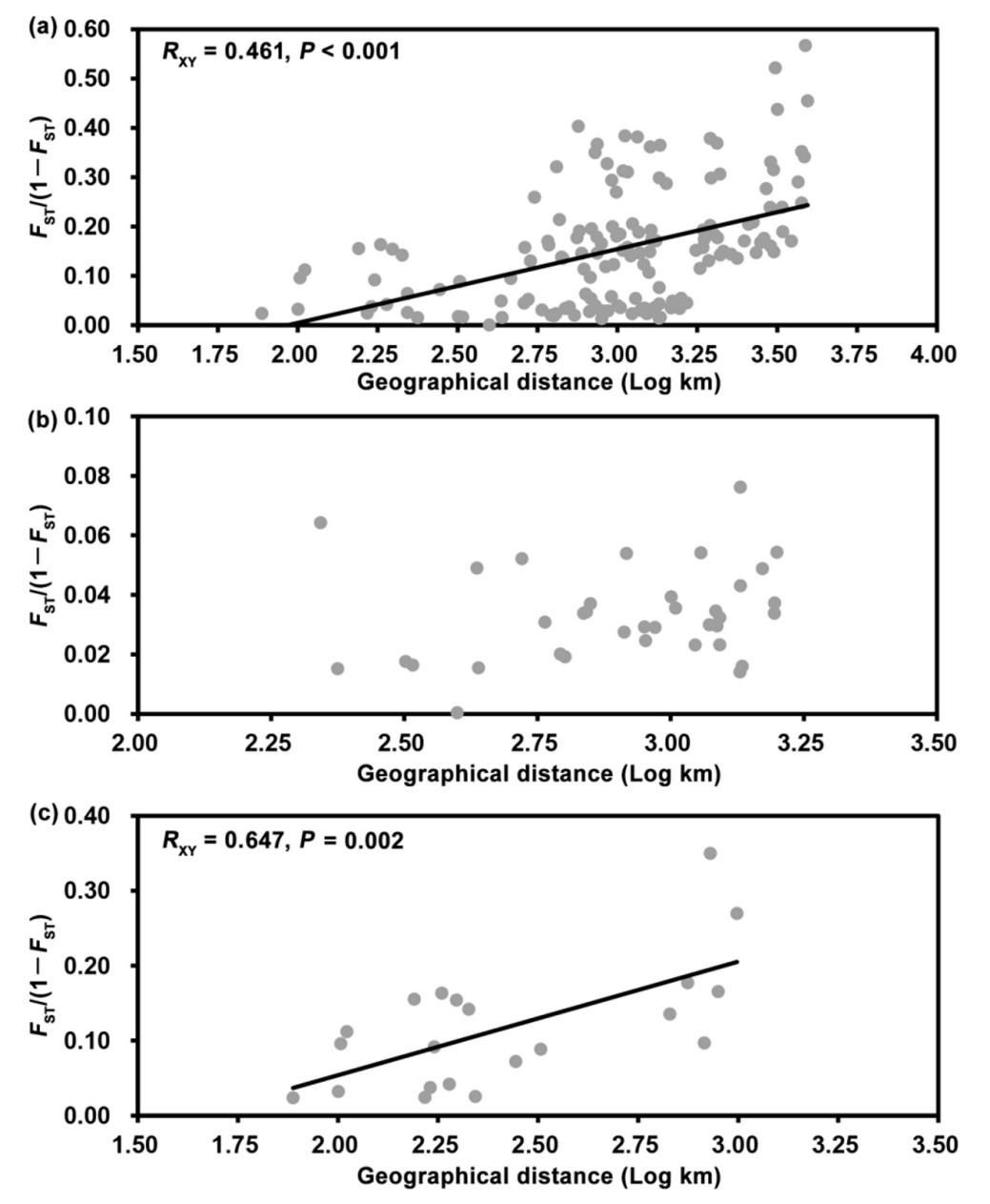


**Fig. S6** Correlation analysis between pairwise *M.* *onukii* populations estimates of linearized *F*_ST_/(1－*F*_ST_) and the logarithms of geographic distance in 18 populations of *M.* *onukii* based on reduced-representation genome sequencing.(a) 18 populations; (b) Cluster 3-1; (c) Cluster 3-2; Cluster 3-1 and Cluster 3-2 were divided by the result of Bayesian clustering analysis when K = 3.


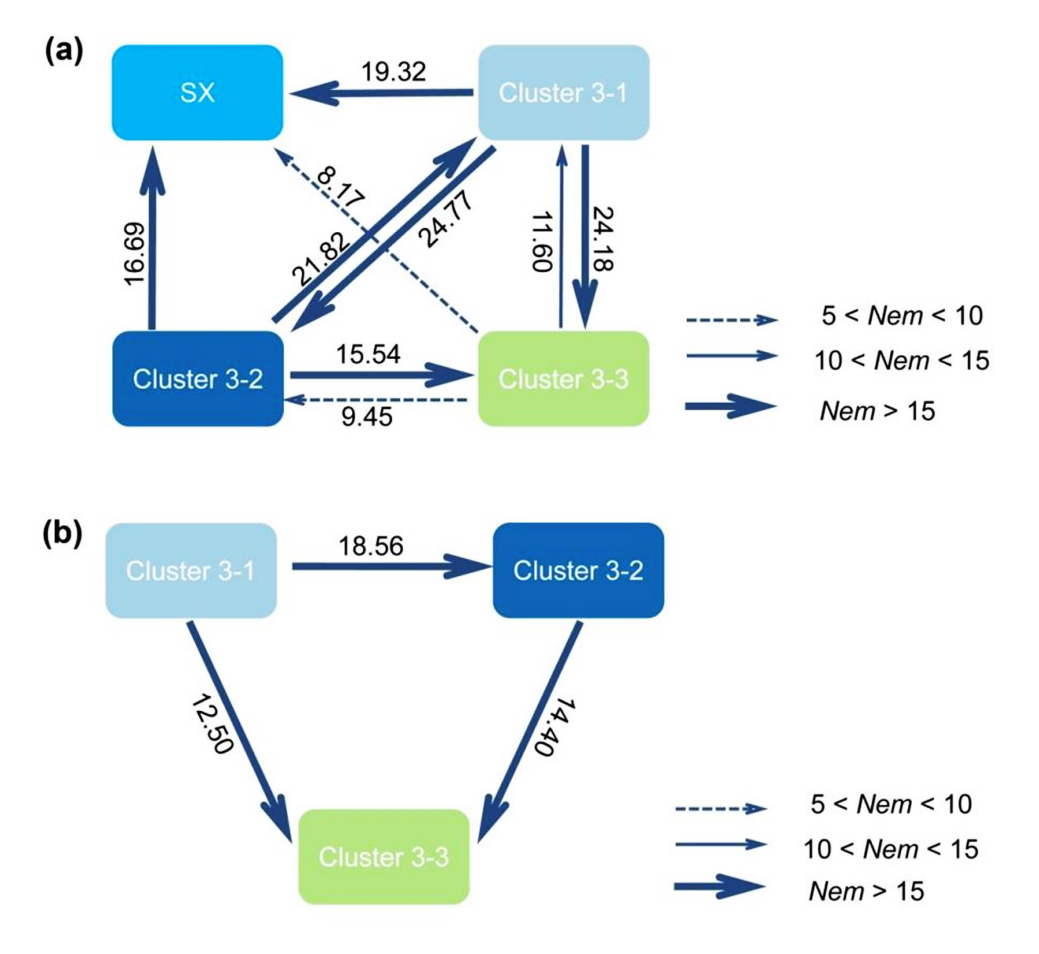


**Fig. S7** Maximum likelihood estimates for gene flow among clusters using SSRs. (a) gene flow among clusters inferred by SSRs. Cluster 3-1 excepted for Hanzhong (SX) populations (included Jinhua (JH), Yingde (YD), Guilin (GL), Fuzhou (FZ), Hainan (HQ) Leshan (CT), Chongqing (CY), Zunyi (ZY) and Vinh Phuc (VN) populations), Cluster 3-2 (Yunnan populations included Chuxiong (CX), Pu’er (PE), Mojiang (MJ), Menghai (MH), Lincang (LX) and Jingdong (JD) populations), Cluster 3-3 (included Kagoshima (JJ) and Shizuoka (JS) populations) and Hanzhong (SX); (b) gene flow among clusters inferred by SNPs, Hanzhong (SX) was included in Cluster 3-1. Ne inside circles is effective population size (i.e *θ* = 4 *Neµ*, where *θ* is the population size parameter and *µ* is mutation rate) and *Nem*: effective number of migrants per generation (i.e *Nem = θM*/4, where M is scaled migration rate per generation) for *M. onukii*. Arrows indicate the gene flow direction among clusters. *Nem* less than 5 and no significant asymmetrical effective migrants not shown.
